# The myosin activator omecamtiv mecarbil improves wall stress in a rat model of chronic aortic regurgitation

**DOI:** 10.14814/phy2.14988

**Published:** 2021-08-17

**Authors:** Bachar El Oumeiri, Philippe van de Borne, Géraldine Hubesch, Antoine Herpain, Filippo Annoni, Pascale Jespers, Constantin Stefanidis, Kathleen Mc Entee, Frédéric Vanden Eynden

**Affiliations:** ^1^ Department of Cardiac Surgery ULB Erasme University Hospital Brussels Belgium; ^2^ Department of Cardiology ULB Erasme University Hospital Brussels Belgium; ^3^ Laboratory of Physiology and Pharmacology ULB Brussels Belgium; ^4^ Department of Intensive Care ULB Erasme University Hospital Brussels Belgium

**Keywords:** aortic regurgitation, omecamtiv mecarbil, overload, wall stress

## Abstract

In patients with chronic aortic regurgitation (AR), excessive preload and afterload increase left ventricle wall stress, leading to left ventricular systolic dysfunction. Thus, the objective of the present study was to evaluate the effects of the myosin activator omecamtiv mecarbil (OM) on left ventricle wall stress in an experimental rat model of severe chronic AR. Forty adult male Wistar rats were randomized into two experimental groups: induction of AR (acute phase) by retrograde puncture (*n* = 34) or a sham intervention (*n* = 6). Rats that survived the acute phase (*n* = 18) were randomized into an OM group (*n* = 8) or a placebo group (*n* = 10). Equal volumes of OM (1.2 mg/kg/h) or placebo (0.9% NaCl) were continuously infused into the femoral vein over 30 min. OM significantly decreased end‐systolic and end‐diastolic and maximum wall stress in this experimental rat model of chronic severe AR (*p* < 0.001) and increased systolic performance assessed by fractional shortening and left ventricle end‐systolic diameter; both *p* < 0.05). These effects were correlated with decreased indices of global cardiac function (cardiac output and stroke volume; *p* < 0.05) but were not inferior to baseline pump indices. Infusion with placebo did not affect global cardiac function but decreased end‐systolic wall stress (*p* < 0.05) and increased systolic performance (all *p* < 0.001). In the sham‐operated (control) group, OM decreased diastolic wall stress (*p* < 0.05). Based on these results, OM had a favorable effect on left ventricle wall stress in an experimental rat model of severe chronic AR.

## Graphical abstract

Aortic regurgitation increase left ventricle wall stress, the objective of this study is to evaluate the effects of omecamtiv mecarbil on left ventricle wall stress.

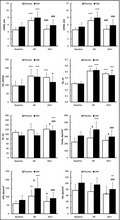

## 1 INTRODUCTION

Aortic regurgitation (AR) is characterized by diastolic reflux of blood from the aorta into the left ventricle (LV) due to incomplete closure of the aortic cusps. The prevalence of chronic AR is not precisely known. The Framingham Offspring study (Singh et al., 1999) reported that the overall prevalence of AR was 13% in men and 8.5% in women.

The central hemodynamic feature of chronic AR is the combined volume and pressure overload of the LV (Carabello, 1990). This results in a series of compensatory mechanisms, including an increase in end‐diastolic volume, an increase in chamber compliance to accommodate the increased volume without an increase in filling pressure, and a combination of eccentric and concentric hypertrophy (Otto & Bonow, 2009). In early compensated severe AR, the LV adapts to the volume overload by eccentric hypertrophy (Ricci, 1982). Over time, progressive LV dilation and systolic hypertension increase wall stress and the volume/mass ratio of the LV (Bekeredjian & Grayburn, 2005). The increase in wall stress leads to overt LV systolic dysfunction, which manifests as a decline in the left ventricle ejection fraction (LVEF; Bekeredjian & Grayburn, 2005), followed by irreversible heart failure and death. Increased LV wall stress has generally been associated with worse clinical outcomes (Greenberg et al., 1985; Kumpuris et al., 1982).

Vasodilator therapy is designed to reduce regurgitant volume, LV volume, and wall stress (Otto & Bonow, 2009). However, in asymptomatic patients with severe AR and normal LV function, vasodilators failed to demonstrate any significant benefit, did not delay the need for surgery, did not reduce regurgitant volume, and had no beneficial effect on LV size or function (Evangelista et al., 2005).

As rats develop LV abnormalities in response to severe AR in a relatively short period (weeks) in contrast to humans (who can tolerate this condition for decades without apparent LV dysfunction), rats make an ideal model to investigate chronic AR (Magid et al., 1984).

Heart failure continues to be a major leading public health problem that is estimated to affect more than 26 million people worldwide; the incidence is steadily increasing, primarily owing to the aging of the population (Ambrosy et al., 2014). The progression of heart failure leads to myocardial contractility deficiency, eventually resulting in a decrease in cardiac output (CO). Although inotropic agents have been shown to improve cardiac contractility, their use has been associated with increased morbidity and mortality caused by intracellular calcium overload, which is associated with ventricular arrhythmia, atrial fibrillation, hypotension, induced myocardial ischemia, increased myocardial oxygen consumption, and direct myocyte toxicity (Tariq & Aronow, 2015; Teerlink et al., 2009). Omecamtiv mecarbil (OM), formerly known as CK‐1827452, is a cardiac myosin activator that increases the proportion of myosin heads bound to actin, creating a force‐producing state that is not associated with cytosolic calcium accumulation (Malik et al., 2011). As the total number of myosin heads bound to actin filaments increases, force production is boosted (Malik et al., 2011). As a result, OM prolongs the duration of total systole by enhancing the rate of entry of myosin into the force‐generating state, which implies increased formation of active cross‐bridges, ultimately leading to stronger cardiac contractions (Liu et al., 2015).

OM increased systolic ejection time and cardiac myocyte fractional shortening in two experimental canine models of heart failure, with no significant increase in LV myocardial oxygen consumption or myocyte intracellular calcium (Shen et al., 2010). One of the main determinants of myocardial oxygen consumption is peak systolic wall stress (Hoffman & Buckberg, 2014). Resting LV myocardial oxygen consumption and wall stress also exhibit a linear relationship, in which doubling wall tension approximately doubles LV oxygen consumption (Strauer, 1979).

In our previous study (El Oumeiri et al., 2018), we showed that OM significantly decreased both LV end‐systolic diameter (LVESD) and LV end‐diastolic diameter (LVEDD) in rats with severe chronic AR. However, we lacked information on preload and afterload, and the anesthesia procedure included intraperitoneal ketamine. Ketamine has a cardiovascular effect resembling sympathetic nervous system stimulation, increasing heart rate and CO (Levänen et al., 1995). In addition, the number of animals in all groups was small, and AR was assessed subjectively using a 1 to 4 scale to classify the severity of regurgitation.

The present study aimed to evaluate the immediate (acute) effects of a single dose of OM on AR and cardiac contractile parameters in an experimental rat model of AR induced by a retrograde puncture. Our model combined the effects of pressure and volume overload on the LV. To determine whether OM affected LV wall stress, the experimental design incorporated both placebo and sham control groups.

## 2 MATERIALS AND METHODS

### 2.1 Experimental animals

The experimental protocol was approved by the Institutional Animal Care and Use Committee of the Free University of Brussels. Studies were conducted in accordance with the *Guide for the Care and Use of Laboratory Animals* published by the National Institutes of Health (NIH Publication No. 85‐23; revised 1996). Forty male adult Wistar rats (486 ± 49 g body weight) were separated into two groups: sham intervention (*n* = 6) or AR induction (*n* = 34). Rats that survived AR induction or the acute phase (*n* = 18) were randomized into the OM (*n* = 8) or placebo (*n* = 10) groups. Rats that underwent the sham intervention (*n* = 6) also received OM and served as controls to assess the effect of time and repeated measurements on the variables investigated in the study.

### 2.2 Anesthesia and surgical procedure

Animals were anesthetized using 1.5% inhaled isoflurane. AR was induced by retrograde puncture of the aortic valve leaflet, as previously described (El Oumeiri et al., 2018). Heart rate (HR) and rhythm were monitored via limb leads throughout the procedure. The right internal carotid artery was surgically exposed and ligated distally; subsequently, a transverse arteriotomy was performed, through which a fixed‐core wire guide (0.025 inch diameter; Cook Inc.) was advanced toward the aortic valve in a retrograde manner to tear the valve leaflets and induce AR. The following echocardiographic criteria after achieving a popping sensation at the time of surgery were used to include animals in the study: (1) a jet extent greater than 30% of the length of the LV, and (2) a color‐Doppler ratio of regurgitant jet width to LV outflow tract diameter greater than 50% (Zoghbi et al., 2003). The six sham‐operated animals underwent cannulation of the right carotid artery without aortic valve puncture. Animals were closely observed during the first hours and days after surgery for any sign of respiratory distress suggestive of acute heart failure. Pre‐ and post‐surgery analgesia were administered.

### 2.3 Cardiac measurements

Transthoracic 2D, M‐mode, and Doppler echocardiography were performed under general anesthesia (1.5% isoflurane) using an ultrasound scanner (Vivid‐E90, GE Healthcare) equipped with a 12‐MHz phased‐array transducer (GE 12S‐D, GE Healthcare). Rats were placed in the right and left lateral recumbent positions, and electrocardiography was conducted via limb leads throughout the procedure. All measurements were made according to the recommendations of the Society of Echocardiography for human subjects (Zoghbi et al., 2003). Standard right parasternal (long‐ and short‐axis) and left apical parasternal views were used for data acquisition. Fractional shortening (FS) was calculated using the formula FS = LVEDD − LVESD/LVEDD × 100, in M‐mode from an LV short‐axis view at the level of the chordae tendineae using the following measured parameters: diastolic (d) and systolic (s) septal wall thickness (SWTd and SWTs, respectively), posterior wall thickness (PWTd and PWTs, respectively), and LV end‐systolic and end‐diastolic diameters (LVEDD and LVESD, respectively). Ejection fraction (EF) was derived using the Teicholz formula. Left ventricle mass was calculated using the American Society of Echocardiography recommended formula: LV mass = 0.8 × {1.04[(LVEDD + PWTd + SWTd)^3^ − (LVEDD)^3^]} + 0.6 g. The aortic diameter was measured from the right long‐axis parasternal view. The aortic flow was measured from the left apical view to calculate forward stroke volume (SV) and CO, and to measure pre‐ejection period (PEP: delay from Q wave of QRS to aortic opening; ms), LV ejection time (LVET: interval from beginning to termination of aortic flow; ms), and interbeat interval (RR; ms). Systolic time (ST; ms) was determined as PEP + LVET (ms), and diastolic time (ms) was calculated as RR interval − systolic time. We also calculated the PEP/LVET ratio, a more useful index of overall LV systolic performance (Lewis et al., 1977), which is better correlated with other LV performance measurements than either PEP or LVET alone and is considered independent of HR (Spodick et al., 1984). The severity of the regurgitated aortic jet was objectively evaluated by measuring the pressure half‐time (PHT) of the AR jet using a continuous‐wave Doppler. A PHT of <200 ms was considered indicative of severe AR. Relative wall thickness (RWT) was calculated using the formula RWT = 2·PWTd/LVEDD, where PWTd is the posterior wall thickness at end‐diastole (mm).

### 2.4 Calculation of wall stress variables

Wall stress was calculated based on Laplace's law (*σ* = *P* × *r*/2*w*, where *σ* is the wall stress, *P* is the left intraventricular pressure, *r* is the LV diameter, and *w* is the wall thickness.) Wall stress—the true measure of LV afterload—decreases during ejection and is twice as high in protosystole as in telesystole. The calculation of maximum stress (*σ*_max_) must be performed at maximum systolic pressure using the telediastolic diameter, which is that of protosystole before it shortens during ejection as follows: *σ*_max_ = (Ps_max_·Dtd)/2*w*, where *P* is the maximum systolic pressure, *D* is the LVEDD, and *w* is the end‐diastolic wall thickness. End‐systolic wall stress (*σ*_es_) is calculated using the formula *σ*_es_ = Pes·Dts/2*w*, where Pes is the end‐systolic pressure, Dts is the LVESD, and w is the end‐systolic wall thickness. Wall stress on diastole (*σ*_d_) was also calculated: *σ*_d_ = Pd·Dtd/2*w*, where Pd is the diastolic pressure.

### 2.5 Invasive blood pressure measurement

Invasive arterial pressures were measured with a micro manometer (rodent catheter 1.6 F, Transonic Systems Inc.) inserted in the right common carotid artery before and after the induction of AR (only once in sham‐operated rats) and the left femoral artery for all rats before and after injection of OM or placebo (2 months after induction of AR). The micro manometer was connected to a data acquisition system (ADV500PV system, Transonic Systems Inc.).

### 2.6 Experimental design

Doppler echocardiography was performed before AR induction (baseline) during surgery to confirm the presence and severity of AR. Doppler echocardiography was performed again 2 months after the induction of AR, both before and after the infusion of OM (1.2 mg/kg/h) or placebo (0.9% NaCl). In the treatment groups, animals received equal volumes (12 ml/kg) of placebo (*n* = 10) or OM (*n* = 8) employing femoral vein perfusion for 30 min. This procedure achieved a plasma concentration of nearly 400 ng of OM/ml in a previous study (Anderson et al., 2005). Doppler echocardiography was performed immediately after the 30‐min infusion. All animals remained alive during these experimental sessions.

### 2.7 Statistical analyses

Results are expressed as means ± standard deviation (SD). Data were analyzed using a two‐factor analysis of variance (ANOVA) for repeated measures. Inter‐group differences were tested using two‐way ANOVA. If the *F* ratio of the ANOVA reached the threshold *p*‐value of <0.05, further comparisons were made using the parametric Student's *t* test. A *p*‐value of <0.05 was considered to be significant (SPSS 23.0, IBM Corp.).

## 3 RESULTS

### 3.1 LV and AR measurements

AR was achieved in all 34 rats, as confirmed by the presence of a regurgitant jet quantified as severe (PHT <200 ms). Sixteen rats died during induction surgery or from congestive heart failure before the end of the 2‐month follow‐up and were thus excluded from the final analysis. After 2 months, AR (PHT <200 ms) was confirmed, and the presence of volume overload and eccentric hypertrophy were established echocardiographically by significant increases in LVEDD, LVESD, and LV mass (*n* = 18, all *p* < 0.001, Figure 2). Load‐dependent indices of LV systolic function (FS and EF) and RWT were significantly lower than baseline (*p* < 0.05, *n* = 18), whereas SV and CO were significantly higher than baseline (*n* = 18, both *p* < 0.001). As expected, signs of AR were not present in the sham‐operated rats (*n* = 6), and thus no changes in LV functions or dimensions were observed in this group.

### 3.2 Measures of wall stress and blood pressure

Diastolic arterial blood pressure was significantly lower after the induction of AR (*n* = 18, *p* < 0.001). End‐systolic and maximum wall stress were significantly higher in all rats with AR after induction (*n* = 18, *p* < 0.05). We detected no changes in *σ*_max_ or arterial blood pressure in sham‐operated rats (*n* = 6).

### 3.3 Effects of placebo in rats with AR

**FIGURE 1:**
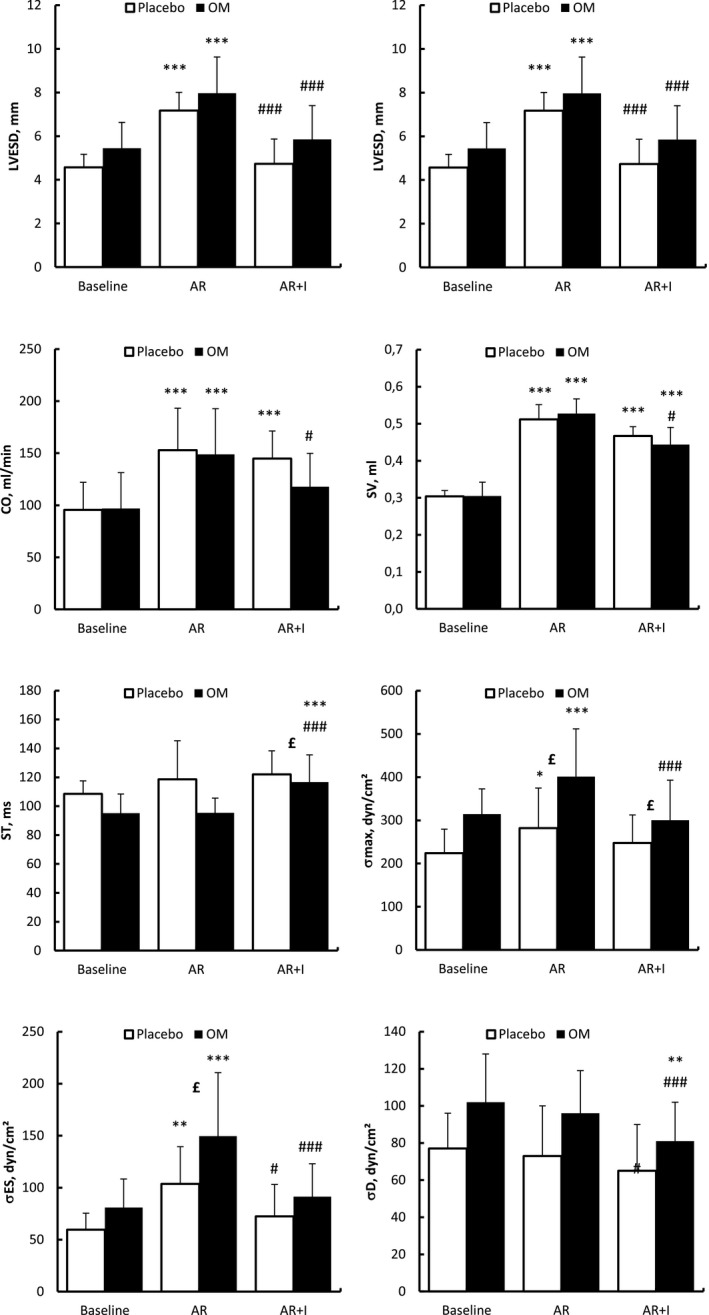
Hemodynamic effects of aortic regurgitation (AR) induced in rats at baseline, 2 months after induction of AR (pre‐infusion), and following infusion (I) of omecamtiv mecarbil (OM) or placebo (0.9% NaCl)^1^. ^1^Values are expressed as mean ± SD. *p < 0.05; **p < 0.01; ***p < 0.001 (or other symbols; two‐way analysis of variance). Comparisons: * = within a group compared with baseline; # = within a group compared with 2 months (pre‐infusion); £ = compared with placebo group at the same time point; # within the same goup (placebo (n=10) or OM (n=8) before and after infusion, the 2 group had a AR; ### is p<0.001 (in the same group placebo after 2 months before and after infusion of placebo n=10, or for the OM group (n=8) after 2 months before and after OM infusion). CO, cardiac output; LVEDD, left ventricle end‐diastolic diameter; LVESD, left ventricle end‐systolic diameter; ST, systolic time; SV, stroke volume; *σ*, max wall stress; *σ*_d_, diastolic wall stress; *σ*_es_, end‐systolic wall stress

Infusion with 0.9% NaCl (placebo group, *n* = 10; Figure 1) affected some echocardiographic parameters in rats with AR. FS was significantly higher (*p* < 0.05), whereas LVESD, LVESDD, and end‐systolic wall stress were significantly lower (*p* < 0.05). Hemodynamically, NaCl infusion significantly increased systolic and diastolic blood pressures (*p* < 0.05), as well as PWTs and the PEP/LVET ratio (*p* < 0.05).

### 3.4 Effects of OM in rats with AR

Infusion with OM (treatment group, *n* = 8; Figure 1; supplement files) significantly increased FS, ST, and LVET (*p* < 0.05) and significantly decreased LVEDD and LVESD (*p* < 0.05). In addition, OM treatment resulted in a significant decrease in the wall stress parameters *σ*_max_, *σ*_es_, and *σ*_d_ (*p* < 0.05). OM infusion also affected indices of global cardiac function, including significant decreases in SV and CO (*p* < 0.05), but did not affect the severity of AR (PHT 110 ± 12 ms vs. 89 ± 10 ms before OM injection, P ns).

### 3.5 Effects of OM compared with placebo in rats with AR

The effects of OM and placebo treatments in AR rats were compared by using two‐way ANOVA (Figure 1, see supplement files). In the comparison of OM versus placebo, values for PEP (*p* < 0.01), the PEP/LVET ratio (*p* < 0.001), the ST/RR ratio (*p* < 0.01), and SWTs (*p* < 0.01) were lower in rats infused with OM than those in the placebo group. Similarly, diastolic time (DT) was greater in rats of the OM group than in rats of the placebo group (*p* < 0.05). No other echocardiographic or hemodynamic parameters investigated in this study exhibited significant differences between the OM and placebo groups.

### 3.6 Effects of OM in sham‐operated rats

The effects of OM treatments in AR rats and sham‐operated rats were compared by two‐way ANOVA (see supplement files). Infusion of OM in sham‐operated rats (*n* = 6) resulted in significant decreases in *σ*_d_, the PEP/LVET ratio, and PEP (all *p* < 0.05).

## 4 DISCUSSION

The main findings of our study are that OM decreased LV wall stress parameters associated with the prolongation of ejection time without improvement of indices of global cardiac function (SV, CO), but with maintenance of baseline global cardiac function in rats with severe chronic AR.

### 4.1 Effect of AR on cardiac function and wall stress

**FIGURE 2:**
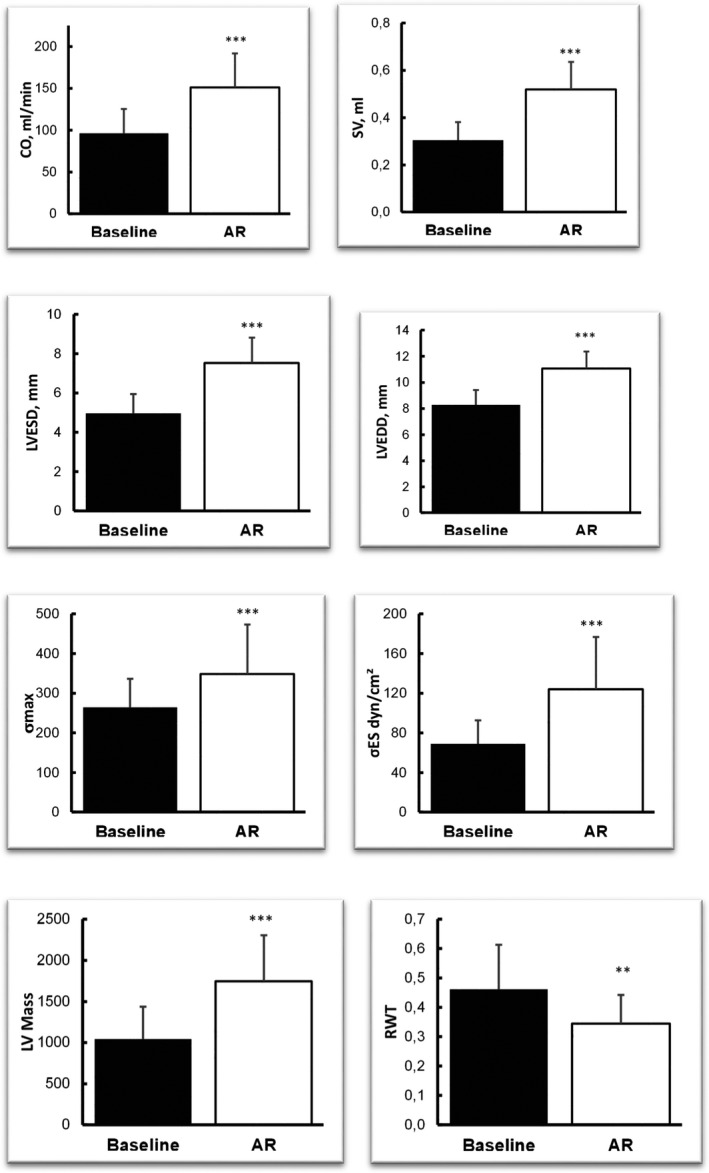
Hemodynamic effects of aortic regurgitation (AR) induced in rats (*n* = 18) at baseline and 2 months after induction of AR. Values are expressed as mean ± SD. *p < 0.05; **p < 0.01; ***p < 0.001 (or other symbols; two‐way analysis of variance). Comparisons: * = within a group compared with baseline; * between the same rats that had an AR 2 months before (n=18), before AR and 2 months after induction of AR. CO, cardiac output; LVEDD, left ventricle end‐diastolic diameter; LVESD, left ventricle end‐systolic diameter; RWT, relative wall thickness; SV, stroke volume; *σ*, max wall stress; *σ*_d_, diastolic wall stress; *σ*_es_, end‐systolic wall stress

Chronic severe AR imposes a combined volume and pressure overload on the LV. The volume overload is a consequence of the regurgitant volume itself (Bekeredjian & Grayburn, 2005), whereas the pressure overload results from systolic hypertension, which occurs as a result of an increase in total aortic SV from both the regurgitant volume and the forward stroke volume that is ejected into the aorta during systole (Bekeredjian & Grayburn, 2005). This effect was observed in the 18 rats with successful induction of AR (Figure 2). In compensated severe AR, eccentric hypertrophy with combined concentric hypertrophy of the LV is an essential adaptive response to volume overload, which itself is a compensatory mechanism that permits the ventricle to normalize its afterload and maintain normal ejection performance (physiologic hypertrophy; Grossman, 1980). This effect was observed in our rat model, as demonstrated by an RWT value of 0.34 ± 0.02 (*n* = 18), with LV dilation, corresponding to physiologic hypertrophy (Gaasch et al., 2011), in agreement with the LV structural remodeling previously described (Gaasch et al., 2011) in humans. Sarcomeres are laid down in series, and myofibers are elongated (Ricci, 1982), and eccentric hypertrophy preserves LV diastolic compliance and increases LV mass, such that the volume/mass ratio is normal, and LVEF is maintained by increased preload (Bekeredjian & Grayburn, 2005). Again, these effects were observed in our model (Figure 2).

LV dilatation and systolic hypertension increase wall stress and volume/mass ratio. In the present study, LV wall stress was elevated in rats with AR, and EF was significantly lower than baseline, 2 months after the induction of AR (Figure 2). Taniguchi et al. (2000) reported an abnormal relationship between EF (depressed contractility) and LV wall stress in patients with chronic AR and advanced cellular hypertrophy which worsened with LV enlargement. Percy et al. (1993) addressed the prognostic significance of LV wall stress in asymptomatic patients with AR, and concluded that elevated wall stress in chronic AR predicts a faster deterioration of LV function. Greenberg et al. (1985) demonstrated associations between EF response and systolic wall stress and concluded that patients with EF decreased during exercise had elevated resting LV systolic wall stress. The groups studied had similar near‐normal LV end‐systolic dimensions and abnormal LV wall stress, suggesting that elevated wall stress in chronic AR predicts a poorer mechanical and clinical prognosis that may be independent of classical parameters such as LV dimensions and LV function.

### 4.2 Effect of OM on wall stress and AR

Our results showed that treatment with OM decreased maximum wall stress, end‐systolic wall stress, and diastolic wall stress of the LV (Figure 1) in our rat model of AR. In our model, this effect was related to a decrease in LVEDD and a decrease in average maximum systolic pressure. End‐systolic wall stress was significantly lower (*p* < 0.05) in the placebo group (*n* = 10), but to a lesser extent than in the OM group (*n* = 8; *p* < 0.001). Furthermore, decreases in LVEDD and end‐diastolic pressure following OM have been reported in animal models of ischemia (Bakkehaug et al., 2015; Rønning et al., 2018). Reducing ventricular wall stress is considered a cornerstone in treating heart failure (Yin, 1981). In its simplest form, as described by Laplace's law, ventricular wall stress is directly proportional to the diameter of the ventricle and ventricular pressure and is inversely proportional to the wall thickness of the ventricle. It is widely believed that increased ventricular wall stress is responsible for the adverse remodeling process that eventually leads to heart failure (Grossman, 1980). Increased wall stress is an independent predictor of subsequent LV remodeling (Hung et al., 2010). One of the main determinants of myocardial oxygen usage is peak systolic wall stress (Hoffman & Buckberg, 2014). Because the cavity decreases in size and the wall thickens during ejection, protosystolic stress is twice the telesystolic stress (Kolev et al., 1995). This variation is greater than that of the pressures during systole; therefore, peripheral vascular resistance overestimates the overload losses secondary to vasodilatation but underestimates increases caused by vasoconstriction (Lang et al., 1986).

In the present study, OM extended LVET and ST, as reported previously (Liu et al., 2015), without changes in DT. OM did not affect the severity of AR, as measured by AR PHT <200 ms. This finding agrees with the results of our previous study (El‐Oumeiri et al., 2018). However, in our previous study, we subjectively graded the severity of the AR jet on a scale from 1 to 4, whereas in this study, we used an objective quantification of AR to measure the effect of OM on AR more precisely.

### 4.3 Effect of OM on cardiac function

OM decreased SV and CO without an effect on HR but with prolongation of LVET and ST (Tables S1–S3). In contrast, OM increased FS in a similar manner to classic inotropes by improved emptying in systole. Furthermore, we did not observe any SV, CO, or HR changes in our placebo group. The extended myocardial systole could have caused the reduced contractile efficiency observed in the OM group. The fact that LVEDD, and consequently the LV volume, decreased despite unchanged preload and HR suggests that OM induces myocardial constraint in late diastole. This is in line with a previous study that reported that OM slows relaxation and increases passive tension at rest in isolated rat cardiomyocytes (Nagy et al., 2015).

Shen et al. (2010) reported that OM significantly increases CO and SV. However, their study differed in several aspects from the present study. They used a canine model of ischemia, the animals were conscious, and the dose of OM and duration of infusion were different. All these factors could explain the difference in the effect of OM on cardiac function compared with our study. Nevertheless, OM significantly extended LVET in both studies.

Because our AR model did not exhibit characteristics of diastolic dysfunction, the interpretation that OM impairs diastolic performance warrants caution. The impairment of diastolic function by OM was reported by Rønning et al. (2018) in pigs with acute ischemic heart failure. OM failed to restore general pump indices such as SV, CO, and EF in the pig model. Likewise, they found no significant changes in SV and CO in the pig model of ischemia in response to OM treatment.

In our previous study (El Oumeiri et al., 2018), OM increased SV and CO in rats with AR. In that study, the animals were anesthetized with an intraperitoneal injection of ketamine/medetomidine. Ketamine is a dissociative anesthetic agent with a cardiovascular effect resembling sympathetic nervous system stimulation, increasing HR and CO (Levanen et al., 1995). Medetomidine improves muscle relaxation, potentiates the anesthetic action of ketamine, and compensates for the cardiac‐stimulating effect of ketamine by decreasing HR and CO. Dexmedetomidine had no direct myocardial depressant effect in the rat heart in doses like those encountered in clinical conditions (Lee et al., 2017). Because the different animal groups were all anesthetized using the same regimen, the decrease in HR we observed can be attributed to OM in our previous study. However, we cannot predict whether this bradycardic effect of OM would also have occurred in conscious, non‐sedated animals. In the current study, animals were anesthetized using 1.5% inhaled isoflurane. Current evidence suggests that isoflurane exerts a negative inotropic effect (Davies et al., 1999). This in contrast with the increase in SV during isoflurane anesthesia we observed in this study, possibly because the negative inotropic effects of isoflurane can be overridden by a decrease in systemic vascular resistance (Heerdt et al., 1998).

## 5 STUDY LIMITATIONS

This study was performed with a limited number of rats with chronic AR and thus represented a narrow observational window into the natural course of this disease. Further investigations with a larger sample size will be necessary for a more robust evaluation, interpretation, and corroboration of our findings. We also did not assess whether OM has dose‐dependent hemodynamic effects in our model of AR. Another limitation is that, although measurements of RWT are applicable in human models, it is unclear whether the same is true of the rat model used in the present study.

## 6 CONCLUSIONS

Our data demonstrate that OM significantly decreased LV wall stress in rats with induced chronic severe AR. In terms of cardiac function, we observed a decrease in SV and CO, but no inferior general pump indices or (in other words) baseline values of SV and CO. Although OM significantly increased the duration of LVET and ST, it did not affect the duration of DT or the severity of AR. The observed effects of OM in the current study reflect the acute (immediate) effect of a single dose; we did not evaluate effects in either the short or medium term.

## CONFLICT OF INTEREST

None declared.

## AUTHOR CONTRIBUTIONS

All experiments were conducted in the Physiology and Pharmacology Laboratory of the University of Brussels. BE conceived the study, participated in its design, carried out the experiments, performed the statistical analysis, and drafted the manuscript. KME conceived and coordinated the study, participated in its design, and carried out the echocardiography. FA and AH read the echocardiography. GH participated in the coordination of the study and carried out the experiments. PJ and CS provided technical assistance, performed the statistical analysis, and reviewed the manuscript. PVB and FV conceived the study and participated in the drafting of the manuscript. All authors read and approved the final manuscript.

## Supporting information



Table S1‐S3
