# Supplementary material for: The myosin activator omecamtiv mecarbil improves wall stress in a rat model of chronic aortic regurgitation
Source: Physiol Rep. 2021 Aug 17;9(16):e14988. doi: 10.14814/phy2.14988 (PMC8371349; doi:10.14814/phy2.14988)
Supplement: Supplementary file 1 — Table S1‐S3 [file PHY2-9-e14988-s001.docx]

Table 1(supplement file). Hemodynamic effects of aortic regurgitation (AR) induced in rats at baseline, 2 months after induction of AR (pre-infusion), and following infusion of omecamtiv mecarbil (OM) or placebo (0.9% NaCl)1

|  | AR rats: Placebo group (n = 10) | | | | | | | | | | | | AR rats: OM group (n = 8) | | | | | | | | | | | | p group | | |
| --- | --- | --- | --- | --- | --- | --- | --- | --- | --- | --- | --- | --- | --- | --- | --- | --- | --- | --- | --- | --- | --- | --- | --- | --- | --- | --- | --- |
| Parameter^2^ | Baseline | | | | 2 mo (pre-infusion) | | | | Post-infusion (NaCl) | | | | Baseline | | | | 2 mo (pre-infusion) | | | | Post-infusion (OM) | | | | Baseline | 2 mo | Post-infusion |
| FS, % | 42 | ± | 5 |  | 33 | ± | 4 | * | 44 | ± | 11 | ### | 38 | ± | 8 |  | 31 | ± | 10 | * | 38 | ± | 10 | # |  |  |  |
| EF, % | 76 | ± | 10 |  | 65 | ± | 11 | ** | 70 | ± | 10 |  | 73 | ± | 11 |  | 63 | ± | 14 | * | 68 | ±4 | 11 |  |  |  |  |
| SWTs, mm | 3.03 | ± | 0.57 |  | 2.85 | ± | 0.69 |  | 3.20 | ± | 0.79 |  | 2.32 | ± | 0.24 |  | 2.38 | ± | 0.32 |  | 2.51 | ± | 0.20 |  | ££ |  | ££ |
| SWTd, mm | 2.20 | ± | 0.56 |  | 2.38 | ± | 0.76 |  | 2.42 | ± | 0.48 |  | 1.62 | ± | 0.28 |  | 1.78 | ± | 0.15 |  | 1.87 | ± | 0.20 |  |  | £ |  |
| LVESD, mm | 4.6 | ± | 0.6 |  | 7.2 | ± | 0.8 | *** | 4.7 | ± | 01.1 | ### | 5.4 | ± | 1.2 |  | 8.0 | ± | 1.7 | *** | 5.9 | ± | 1.5 | ### |  |  |  |
| LVEDD, mm | 7.9 | ± | 0.6 |  | 10.7 | ± | 1.1 | *** | 8.5 | ± | 0.8 | ### | 8.8 | ± | 1.4 |  | 11.6 | ± | 1.6 | *** | 9.4 | ± | 2.1 | ### |  |  |  |
| HR, bpm | 315 | ± | 61 |  | 301 | ± | 46 |  | 313 | ± | 40 |  | 301 | ± | 36 |  | 279 | ± | 39 |  | 273 | ± | 45 |  |  |  |  |
| LVOT, mm | 2.41 | ± | 0.19 |  | 2.82 | ± | 0.19 | *** | 2.85 | ± | 0.16 | *** | 2.58 | ± | 0.13 |  | 2.69 | ± | 0.15 |  | 2.69 | ± | 0.20 |  |  |  |  |
| PWTs, mm | 3.00 | ± | 0.46 |  | 2.89 | ± | 0.76 |  | 3.45 | ± | 0.61 | *# | 2.69 | ± | 0.43 |  | 2.85 | ± | 0.65 |  | 2.93 | ± | 0.51 |  |  |  |  |
| PWTd, mm | 1.88 | ± | 0.53 |  | 1.76 | ± | 0.46 |  | 2.35 | ± | 0.50 |  | 1.82 | ± | 0.35 |  | 2.01 | ± | 0.30 |  | 2.06 | ± | 0.43 |  |  |  |  |
| PEP, ms | 28 | ± | 6 |  | 31 | ± | 9 |  | 34 | ± | 9 | ** | 20 | ± | 10 |  | 12 | ± | 7 | ** | 15 | ± | 8 |  |  | ££ | ££ |
| LVET, ms | 81 | ± | 4 |  | 87 | ± | 8 |  | 88 | ± | 12 |  | 76 | ± | 6 |  | 84 | ± | 9 |  | 100 | ± | 29 | ***## |  |  |  |
| ST, ms | 109 | ± | 9 |  | 119 | ± | 14 |  | 122 | ± | 19 |  | 95 | ± | 13 |  | 95 | ± | 10 |  | 117 | ± | 32 | ***### |  | £ |  |
| DT, ms | 88 | ± | 28 |  | 85 | ± | 27 |  | 72 | ± | 16 |  | 112 | ± | 24 |  | 121 | ± | 21 |  | 115 | ± | 19 |  |  | £ | £ |
| RR, ms | 197 | ± | 34 |  | 202 | ± | 30 |  | 194 | ± | 26 |  | 206 | ± | 26 |  | 216 | ± | 26 |  | 232 | ± | 40 |  |  |  |  |
| PEP/LVET | 0.34 | ± | 0.06 |  | 0.36 | ± | 0.11 |  | 0.38 | ± | 0.09 | ## | 0.25 | ± | 0.12 |  | 0.13 | ± | 0.09 | *** | 0.15 | ± | 0.07 | ** |  | £££ | £££ |
| ST/RR | 0.58 | ± | 0.11 |  | 0.59 | ± | 0.08 |  | 0.63 | ± | 0.07 |  | 0.46 | ± | 0.06 |  | 0.44 | ± | 0.04 |  | 0.50 | ± | 0.07 |  | ££ | £££ | ££ |
| ARPht, ms |  |  |  |  | 92 | ± | 24 |  | 123 | ± | 49 | # |  |  |  |  | 89 | ± | 28 |  | 110 | ± | 30 |  |  |  |  |
| SV, ml | 0.30 | ± | 0.05 |  | 0.51 | ± | 0.13 | *** | 0.47 | ± | 0.08 | *** | 0.31 | ± | 0.11 |  | 0.53 | ± | 0.11 | *** | 0.44 | ± | 0.13 | ***# |  |  |  |
| CO, ml/min | 96 | ± | 26 |  | 153 | ± | 40 | *** | 145 | ± | 27 | *** | 97 | ± | 35 |  | 149 | ± | 44 | *** | 118 | ± | 32 | # |  |  |  |
| LVOT VTI, mm | 66 | ± | 12 |  | 81 | ± | 11 |  | 74 | ± | 13 |  | 63 | ± | 14 |  | 94 | ± | 17 |  | 77 | ± | 19 |  |  |  |  |
| BP Sys, mmHg | 119 | ± | 5 |  | 115 | ± | 7 |  | 135 | ± | 12 | **### | 114 | ± | 6 |  | 131 | ± | 19 | ** | 120 | ± | 12 |  |  |  |  |
| BP Dia, mmHg | 82 | ± | 3 |  | 59 | ± | 10 | *** | 69 | ± | 13 | **# | 73 | ± | 7 |  | 62 | ± | 10 | * | 56 | ± | 9 | *** |  |  |  |
| Weight, g | 502 | ± | 84 |  | 577 | ± | 75 | *** |  |  |  |  | 466 | ± | 64 |  | 544 | ± | 52 | *** |  |  |  |  |  |  |  |
| LV Mass | 1094 | ± | 442 |  | 1750 | ± | 661 | *** | 1453 | ± | 385 | # | 978 | ± | 343 |  | 1742 | ± | 441 | *** | 1328 | ± | 577 | *# |  |  |  |
| σd, dyn/cm² | 77 | ± | 19 |  | 73 | ± | 27 |  | 65 | ± | 25 |  | 102 | ± | 26 |  | 96 | ± | 23 |  | 81 | ± | 21 | **### |  | £ |  |
| σmax, dyn/cm² | 224 | ± | 56 |  | 282 | ± | 93 | * | 248 | ± | 65 |  | 314 | ± | 59 |  | 401 | ± | 111 | *** | 300 | ± | 93 | ### | £ | ££ |  |
| σEs dyn/cm² | 60 | ± | 16 |  | 104 | ± | 36 | ** | 73 | ± | 31 | # | 81 | ± | 28 |  | 150 | ± | 61 | *** | 91 | ± | 32 | ### |  | £ |  |
| RWT | 0.49 | ± | 0.17 |  | 0.33 | ± | 0.10 | ** | 0.56 | ± | 0.16 | ## | 0.43 | ± | 0.13 |  | 0.36 | ± | 0.10 |  | 0.46 | ± | 0.14 |  |  |  |  |

1Values are expressed as mean ± SD. *p < 0.05; **p < 0.01; ***p < 0.001 (or other symbols; two-way ANOVA). Comparisons: * = within a group compared with baseline; # = within a group compared with 2 months (pre-infusion); £ = compared with placebo group at the same time point.

2FS, fractional shortening; EF, ejection fraction; SWTs, septal wall thickness in systole; SWTd, septal wall thickness in diastole; LVESD, left ventricle end-systolic diameter; LVEDD, left ventricle end-diastolic diameter; HR, heart rate; LVOT, left ventricle outflow tract diameter; PWTs, posterior wall thickness in systole; PWTd, posterior wall thickness in diastole; PEP, pre-ejection period; LVET, left ventricle ejection time; ST, systolic time; DT, diastolic time; RR, interval between successive R; ARPht, aortic regurgitation pressure half-time; SV, stroke volume; CO, cardiac output; VTI, velocity-time integral; BP, blood pressure; σd, diastolic wall stress; σ, max wall stress; σEs, end-systolic wall stress; RWT, relative wall thickness.

Table 2. (supplement file) Hemodynamic effects of aortic regurgitation (AR) induced in rats at baseline and 2 months after induction of AR compared with sham-operated rats¹

|  | Sham-operated rats (n = 6) | | | | | | | AR rats (n = 18) | | | | | | | | p group | |
| --- | --- | --- | --- | --- | --- | --- | --- | --- | --- | --- | --- | --- | --- | --- | --- | --- | --- |
| Parameter^2^ | Baseline | | | 2 mo | | | | Baseline | | | | 2 mo | | | | Baseline | 2 mo |
| FS, % | 40 | ± | 4 | 39 | ± | 4 |  | 40 | ± | 6 |  | 32 | ± | 7 | *** |  |  |
| EF, % | 74 | ± | 7 | 76 | ± | 4 |  | 75 | ± | 10 |  | 64 | ± | 12 | *** |  |  |
| SWTs, mm | 2.54 | ± | 0.27 | 2.56 | ± | 0.22 |  | 2.72 | ± | 0.57 |  | 2.64 | ± | 0.59 |  |  |  |
| SWTd, mm | 1.65 | ± | 0.11 | 1.68 | ± | 0.17 |  | 1.94 | ± | 0.53 |  | 2.11 | ± | 0.64 |  |  |  |
| LVESD, mm | 5.4 | ± | 0.6 | 5.5 | ± | 0.5 |  | 5.0 | ± | 1.0 |  | 7.5 | ± | 1.3 | *** |  | $ |
| LVEDD, mm | 8.9 | ± | 0.6 | 9.0 | ± | 0.4 |  | 8.3 | ± | 1.1 |  | 11.1 | ± | 1.3 | *** |  | $$ |
| HR, BPM | 285 | ± | 47 | 283 | ± | 57 |  | 309 | ± | 50 |  | 291 | ± | 43 |  |  |  |
| LVOT, mm | 2.37 | ± | 0.29 | 2.54 | ± | 0.19 | * | 2.48 | ± | 0.18 |  | 2.76 | ± | 0.18 | *** |  |  |
| PWTs, mm | 2.49 | ± | 0.11 | 2.63 | ± | 0.12 |  | 2.86 | ± | 0.46 |  | 2.87 | ± | 0.69 |  |  |  |
| PWTd, mm | 1.72 | ± | 0.13 | 1.64 | ± | 0.11 |  | 1.85 | ± | 0.45 |  | 1.87 | ± | 0.41 |  |  |  |
| PEP, ms | 21 | ± | 8 | 21 | ± | 5 |  | 24 | ± | 9 |  | 23 | ± | 13 |  |  |  |
| LVET, ms | 81 | ± | 8 | 79 | ± | 6 |  | 78 | ± | 5 |  | 86 | ± | 8 |  |  |  |
| ST, ms | 103 | ± | 13 | 100 | ± | 8 |  | 103 | ± | 13 |  | 108 | ± | 17 |  |  |  |
| DT, ms | 112 | ± | 22 | 114 | ± | 27 |  | 99 | ± | 28 |  | 101 | ± | 30 |  |  |  |
| RR, ms | 215 | ± | 34 | 214 | ± | 34 |  | 201 | ± | 30 |  | 208 | ± | 28 |  |  |  |
| PEP/LVET | 0.25 | ± | 0.08 | 0.26 | ± | 0.06 |  | 0.30 | ± | 0.10 |  | 0.26 | ± | 0.15 |  |  |  |
| ST/RR | 0.48 | ± | 0.03 | 0.47 | ± | 0.05 |  | 0.53 | ± | 0.11 |  | 0.52 | ± | 0.10 |  |  |  |
| ARPht, ms |  |  |  |  |  |  |  |  |  |  |  | 91 | ± | 25 |  |  |  |
| SV, ml | 0.29 | ± | 0.07 | 0.32 | ± | 0.06 |  | 0.30 | ± | 0.08 |  | 0.52 | ± | 0.12 | *** |  | $$ |
| CO, ml/min | 83 | ± | 24 | 90 | ± | 21 |  | 96 | ± | 29 |  | 151 | ± | 41 | *** |  | $$ |
| LVOT VTI, mm | 67 | ± | 13 | 64 | ± | 11 |  | 65 | ± | 13 |  | 87 | ± | 15 | *** |  |  |
| BP Sys, mmHg | 124 | ± | 19 | 128 | ± | 9 |  | 116 | ± | 6 |  | 122 | ± | 15 |  |  |  |
| BP Dia, mmHg | 88 | ± | 16 | 90 | ± | 10 |  | 78 | ± | 7 |  | 60 | ± | 10 | *** |  | $$$ |
| Weight, g | 479 | ± | 37 | 553 | ± | 38 | *** | 486 | ± | 76 |  | 562 | ± | 66 | *** |  |  |
| LV Mass | 756 | ± | 304 | 996 | ± | 169 |  | 1854 | ± | 675 |  | 1747 | ± | 558 | *** |  | $$ |
| σd, dyn/cm² | 119 | ± | 20 | 126 | ± | 29 |  | 88 | ± | 25 |  | 87 | ± | 30 |  |  | $$ |
| σmax, dyn/cm² | 336 | ± | 51 | 343 | ± | 29 |  | 264 | ± | 72 |  | 348 | ± | 125 | *** |  |  |
| σEs,dyn/cm² | 106 | ± | 28 | 110 | ± | 16 |  | 69 | ± | 24 |  | 124 | ± | 52 | *** |  |  |
| RWT | 0.39 | ± | 0.04 | 0.36 |  | 0.04 |  | 0.46 | ± | 0.15 |  | 0.34 | ± | 0.10 | ** |  |  |

¹Values are expressed as mean ± SD. *p < 0.05; **p < 0.01; ***p < 0.001 (or other symbols; two-way ANOVA). Comparisons: * = within a group compared with baseline; $ = compared with sham-operated rats at the same time point.

²FS, fractional shortening; EF, ejection fraction; SWTs, septal wall thickness in systole; SWTd, septal wall thickness in diastole; LVESD, left ventricle end-systolic diameter; LVEDD, left ventricle end-diastolic diameter; HR, heart rate; LVOT, left ventricle outflow tract diameter; PWTs, posterior wall thickness in systole; PWTd, posterior wall thickness in diastole; PEP, pre-ejection period; LVET, left ventricle ejection time; ST, systolic time; DT, diastolic time; RR, interval between successive R; ARPht, aortic regurgitation pressure half-time; SV, stroke volume; CO, cardiac output; VTI, velocity-time integral; BP, blood pressure; σd, diastolic wall stress; σ, max wall stress; σEs, end-systolic wall stress; RWT, relative wall thickness.

Table 3.(supplement file) Comparative effects of omecamtiv mecarbil (OM) in rats with aortic regurgitation (AR) and sham-operated rats 2 months after operation¹

|  | Sham-operated rats (n = 6) | | | | | | | | | | | AR rats: OM group (n = 8) | | | | | | | | | | | | p group | | |
| --- | --- | --- | --- | --- | --- | --- | --- | --- | --- | --- | --- | --- | --- | --- | --- | --- | --- | --- | --- | --- | --- | --- | --- | --- | --- | --- |
| Parameter^2^ | Baseline | | | 2 mo | | | | Post-infusion (OM) | | | | Baseline | | | | 2 mo | | | | Post-infusion (OM) | | | | Baseline | 2 mo | Post-infusion |
| FS, % | 40 | ± | 4 | 39 | ± | 4 |  | 44 | ± | 4 |  | 38 | ± | 8 |  | 31 | ± | 10 | * | 38 | ± | 10 | # |  |  |  |
| EF, % | 74 | ± | 7 | 76 | ± | 4 |  | 81 | ± | 5 |  | 73 | ± | 11 |  | 63 | ± | 14 | * | 68 | ±4 | 11 |  |  |  |  |
| SWTs, mm | 2.54 | ± | 0.27 | 2.56 | ± | 0.22 |  | 2.73 | ± | 0.31 |  | 2.32 | ± | 0.24 |  | 2.38 | ± | 0.32 |  | 2.51 | ± | 0.20 |  | $$ |  | $$ |
| SWTd, mm | 1.65 | ± | 0.11 | 1.68 | ± | 0.17 |  | 1.77 | ± | 0.23 |  | 1.62 | ± | 0.28 |  | 1.78 | ± | 0.15 |  | 1.87 | ± | 0.20 |  |  | $ |  |
| LVESD, mm | 5.4 | ± | 0.6 | 5.5 | ± | 0.5 |  | 4.6 | ± | 0.9 |  | 5.4 | ± | 1.2 |  | 8.0 | ± | 1.7 | *** | 5.9 | ± | 1.5 | ### |  |  |  |
| LVEDD, mm | 8.9 | ± | 0.6 | 9.0 | ± | 0.4 |  | 8.3 | ± | 1.0 |  | 8.8 | ± | 1.4 |  | 11.6 | ± | 1.6 | *** | 9.4 | ± | 2.1 | ### |  |  |  |
| HR, BPM | 285 | ± | 47 | 283 | ± | 57 |  | 304 | ± | 68 |  | 301 | ± | 36 |  | 279 | ± | 39 |  | 273 | ± | 45 |  |  |  |  |
| LVOT, mm | 2.37 | ± | 0.29 | 2.54 | ± | 0.19 | * | 2.59 | ± | 0.19 | ** | 2.58 | ± | 0.13 |  | 2.69 | ± | 0.15 |  | 2.69 | ± | 0.20 |  |  |  |  |
| PWTs, mm | 2.49 | ± | 0.11 | 2.63 | ± | 0.12 |  | 2.77 | ± | 0.22 |  | 2.69 | ± | 0.43 |  | 2.85 | ± | 0.65 |  | 2.93 | ± | 0.51 |  |  |  |  |
| Pd, mm | 1.72 | ± | 0.13 | 1.64 | ± | 0.11 |  | 1.68 | ± | 0.11 |  | 1.82 | ± | 0.35 |  | 2.01 | ± | 0.30 |  | 2.06 | ± | 0.43 |  |  |  |  |
| PEP, ms | 21 | ± | 8 | 21 | ± | 5 |  | 14 | ± | 7 | *# | 20 | ± | 10 |  | 12 | ± | 7 | ** | 15 | ± | 8 |  |  | $$ | $$ |
| LVET, ms | 81 | ± | 8 | 79 | ± | 6 |  | 88 | ± | 8 |  | 76 | ± | 6 |  | 84 | ± | 9 |  | 100 | ± | 29 | ***## |  |  |  |
| ST, ms | 103 | ± | 13 | 100 | ± | 8 |  | 102 | ± | 14 |  | 95 | ± | 13 |  | 95 | ± | 10 |  | 117 | ± | 32 | ***### |  | $ |  |
| DT, ms | 112 | ± | 22 | 114 | ± | 27 |  | 106 | ± | 43 |  | 112 | ± | 24 |  | 121 | ± | 21 |  | 115 | ± | 19 |  |  | $ | $ |
| RR, ms | 215 | ± | 34 | 214 | ± | 34 |  | 209 | ± | 56 |  | 206 | ± | 26 |  | 216 | ± | 26 |  | 232 | ± | 40 |  |  |  |  |
| PEP/LVET | 0.25 | ± | 0.08 | 0.26 | ± | 0.06 |  | 0.15 | ± | 0.07 | **## | 0.25 | ± | 0.12 |  | 0.13 | ± | 0.09 | *** | 0.15 | ± | 0.07 | ** |  | $$$ | $$$ |
| ST/RR | 0.48 | ± | 0.03 | 0.47 | ± | 0.05 |  | 0.50 | ± | 0.06 |  | 0.46 | ± | 0.06 |  | 0.44 | ± | 0.04 |  | 0.50 | ± | 0.07 |  | $$ | $$$ | $$ |
| ARPht, ms |  |  |  |  |  |  |  |  |  |  |  |  |  |  |  | 89 | ± | 28 |  | 110 | ± | 30 |  |  |  |  |
| SV, ml | 0.29 | ± | 0.07 | 0.32 | ± | 0.06 |  | 0.35 | ± | 0.04 |  | 0.31 | ± | 0.11 |  | 0.53 | ± | 0.11 | *** | 0.44 | ± | 0.13 | ***# |  |  |  |
| CO, ml/min | 83 | ± | 24 | 90 | ± | 21 |  | 106 | ± | 22 |  | 97 | ± | 35 |  | 149 | ± | 44 | *** | 118 | ± | 32 | # |  |  |  |
| LVOT VTI, mm | 67 | ± | 13 | 64 | ± | 11 |  | 67 | ± | 3 |  | 63 | ± | 14 |  | 94 | ± | 17 |  | 77 | ± | 19 |  |  |  |  |
| BP Sys, mmHg | 124 | ± | 19 | 128 | ± | 9 |  | 128 | ± | 9 |  | 114 | ± | 6 |  | 131 | ± | 19 | ** | 120 | ± | 12 |  |  |  |  |
| BP Dia, mmHg | 88 | ± | 16 | 90 | ± | 10 |  | 88 | ± | 8 |  | 73 | ± | 7 |  | 62 | ± | 10 | * | 56 | ± | 9 | *** |  |  |  |
| Poids, g | 479 | ± | 37 | 553 | ± | 38 | *** |  |  |  |  | 466 | ± | 64 |  | 544 | ± | 52 | *** |  |  |  |  |  |  |  |
| LV Mass | 756 | ± | 304 | 996 | ± | 169 |  | 883 | ± | 150 |  | 978 | ± | 343 |  | 1742 | ± | 441 | *** | 1328 | ± | 577 | *# |  |  |  |
| σd, dyn/cm² | 119 | ± | 20 | 126 | ± | 29 |  | 104 | ± | 20 | # | 102 | ± | 26 |  | 96 | ± | 23 |  | 81 | ± | 21 | **### |  | $ |  |
| σmax, dyn/cm² | 336 | ± | 51 | 343 | ± | 29 |  | 306 | ± | 72 |  | 314 | ± | 59 |  | 401 | ± | 111 | *** | 300 | ± | 93 | ### | $ | $$ |  |
| σEs | 106 | ± | 28 | 110 | ± | 16 |  | 86 | ± | 21 |  | 81 | ± | 28 |  | 150 | ± | 61 | *** | 91 | ± | 32 | ### |  | $ |  |
| RWT | 0.39 | ± | 0.04 | 0.36 |  | 0.04 |  | 0.41 | ± | 0.06 |  | 0.43 | ± | 0.13 |  | 0.36 | ± | 0.10 |  | 0.46 | ± | 0.14 |  |  |  |  |

### ¹Values are expressed as mean ± SD. *p < 0.05; **p < 0.01; ***p < 0.001 (or other symbols; two-way ANOVA). Comparisons: * = within a group compared with baseline; # = within a group compared with 2 months (pre-infusion); $ = compared with sham-operated rats at the same time point.

²FS, fractional shortening; EF, ejection fraction; SWTs, septal wall thickness in systole; SWTd, septal wall thickness in diastole; LVESD, left ventricle end-systolic diameter; LVEDD, left ventricle end-diastolic diameter; HR, heart rate; LVOT, left ventricle outflow tract diameter; PWTs, posterior wall thickness in systole; PWTd, posterior wall thickness in diastole; PEP, pre-ejection period; LVET, left ventricle ejection time; ST, systolic time; DT, diastolic time; RR, interval between successive R; ARPht, aortic regurgitation pressure half-time; SV, stroke volume; CO, cardiac output; VTI, velocity-time integral; BP, blood pressure; σd, diastolic wall stress; σ, max wall stress; σEs, end-systolic wall stress; RWT, relative wall thickness.
